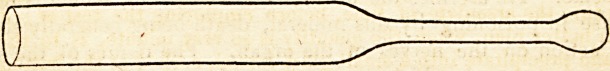# Dr. Christison on Poisoning by Arsenic
*Dr. Christison. Ed. Medico-Chirurgical Transactions, Vol. ii. 1826


**Published:** 1827-10

**Authors:** 


### *Dr. Christison on Poisoning by Arsenic.**

NOTWITHSTANDING what has been written on this important subject, Dr. Christison thinks "that something still remains to be done to complete our knowledge of it." He considers that chemistry has arrived at much certainty, in the great majority of instances, as to the presence of arsenic in the most complex mixtures, although in their quantities exceedingly minute. He thinks, also, that the *symptoms* may sometimes enable us to give a very strong, if not decided opinion, in cases of poisoning by arsenic. His paper is divided into two parts—1st, a history of two cases where arsenic was detected by chemical analysis, in very minute quantity—2dly, comments on the symptomatology of poisoning, and the evidence resting thereon. We shall greatly abridge both these parts of Dr. Christison's paper.

I. The liquid tests for arsenic have been found liable to many fallacies, and their details have seldom carried conviction to the minds of Judge or Jury in this country. It would therefore be desirable, as Dr. C. observes, could the evidence derived from reduction and sublimation be made more delicate than that derived from the use of the liquid tests. It has been proved by Dr. C. himself, as well as by several Continental chemists, that when arsenic is dissolved along with a certain proportion (such as is usually found in medico-legal inquiries) of animal or vegetable matters, not one of the liquid tests can be relied upon. On this account, the whole attention of the medical jurist should be turned, Dr. C. thinks, to the improvement of the analysis by reduction—or at least that the liquid tests should always be subsidiary to the metallization or oxidation of the poison. In the Edinburgh Journal for July, 1824, Dr. C. has published a long paper on arsenic, and endeavoured to shew that so small a quantity as the fourth part of a grain might be presented in its metallic form, although it had been dissolved in eight thousand parts of the most complicated vegetable and animal fluids. The same process was applied to the detection of arsenic in the solid animal tissues, as in the coats of the stomach. The fluids in

---

* Dr. Christison. Ed. Medico-Chirurgical Transactions, Vol. ii. 1826.

which our author detected arsenic, in the above-mentioned proportion, were port wine, porter, tea, with milk, sugar, &c. coffee made in the same manner, barley broth, &c. At that time Dr. C. had not applied his process to the coats of the stomach, which he now does. This brings us to the cases, on which the paper is based.

*Case* 1. T. S. died after somewhat less than two days illness. His wife had lived on bad terms with him, for some time previously, and she frequently threatened vengeance against him. Two or three days before his death, this amiable help-mate went to a schoolmaster, who practised physic a little, and asked for some poison to kill a dog, that had invaded her premises sometimes. The simple schoolmaster, believing her story, offered her some nux vomica, but this did not suit her purpose, and she obtained half a drachm of arsenic. She never, according to her own account, laid it for the dog—indeed, there was no dog but her husband, to lay it for. After the funeral of the husband, the schoolmaster began to feel some qualms of conscience, and sent to the widow for the arsenic, if it had not been used. She had not given it to the dog, and yet she could not produce it. A week after this, the widow applied to the schoolmaster for a medicine of a very different character—namely a love-powder, for one of the male sex, viz: a lad belonging to a smack! The medical history of the case is defective, with regard to the symptoms. The patient was liable to periodical attacks of colic, attended, it was said, with sickness and vomiting. He went to work on a Friday morning, rather indisposed, and was seen returning home in the evening, apparently in good health. On Saturday morning he was decidedly ill—could take no breakfast—complained of head-ache, pain in the breast and belly—vomiting—thirst, &c. In the evening he was seen by a neighbour straining violently—the same on Sunday morning, but he was then very low, and scarcely able to speak. At 3 that day, he felt easier—in the evening he fell down out of his chair, and expired. The whole illness lasted about 36 hours. The body was buried three days after death; and fourteen days after sepulture it was disinterred for examination. We shall quote the *proces-verbal* of the report, as a specimen not very favourable, in a medico-legal point of view.

" ' *External Appearances.* The integuments covering the lower part of the neck, and superior portion of the sternum, were of a dark-red appearance; that covering the abdomen, and more especially on the right and left sides, were of a leaden hue,—little or no perceptible change on the lower extremities.

" ' *Internal Appearances.* The whole outer surface of the stomach was highly vascular, and of a dark livid colour, bearing strong marks of recent and violent inflammation in that viscus, and containing about three or four ounces of fluid.

" ' The small intestines were covered with a tinge of pale red, but in no part assuming the dark livid appearance as exhibited in the outer coat of the stomach. The large intestines were of a healthy aspect, as were also the other viscera of that cavity.

"'Having neither the proper tests, nor the conveniency on the Island, for analyzing the suspected fluid, I postponed the examination of the inner surface of the stomach and intestines, until my return to K——, where, assisted by Dr.—— and Mr.——, the inner coat of the stomach was found streaked with broad patches of a dark livid colour, and interspersed with yellow spots of a golden appearance; and, at the part where the inflammation seemed to be most violent, there was an irregular figure or ring, about a line in breadth, and an inch in circumference, of the same golden appearance, firmly adhering to its inner coat; but no abrasion or ulceration in any part of the stomach or intestines, nor any unnatural appearance in the latter, unless a slight enlargement or fulness of some of their bloodvessels.

"'Although seventeen days had intervened from the time of his death to the day on which the body was examined, yet, from the comparatively slight degree of decomposition that had taken place, as well as from the great contrast between the morbid appearances of the stomach and intestines, there can be no hesitation in saying, that violent inflammation of the former may be considered as the immediate cause of death.

"'*—— *—— *Surgeon.*'"

The above report is much better, Dr. C. informs us, than the average of a great number which he has collected, and certainly shews that pathological investigations are very slovenly conducted, in too many instances, in these Islands. It is to be accounted for, in part, to the little weight attached to medical evidence by our courts of law.

"'They generally contain a meagre statement of facts,—are frequently defective, in so far as the whole organs have not been examined; are commonly couched in ambiguous and inelegant language; sometimes intermeddle with the moral proof: very often give nothing more than an opinion, with one or two facts on which it is grounded; and not unfrequently the opinion, in relation to the facts, is an absolute *non sequitur*. The worst of this practice is, that not only the inspectors do not supply themselves with the proper grounds of judgment; but likewise persons at the head of the profession, to whom their report is submitted, and whose opinion often, perhaps generally, decides the case, are left without the means of judging, till they enter the witness-box; and too frequently cannot form a judgment even there, for want of sufficient data." 285.

The foregoing report is defective in many respects. The head was not opened, nor even the chest. The throat and gullet were not examined, where pathological evidence of poison would be likely to be found. In the description of the outer coat of the stomach, the surgeon states an opinion, where he ought to have confined himself to facts—and possibly that opinion, as to inflammation, was wrong. Inflammation of the peritoneal covering of the stomach is rare in poisoning by arsenic. He ascribes the death to inflammation, which is not often the case in poisoning by this mineral, death being generally the result of its action on the nerves of the organ. The tissues of the stomach were not examined, which was a great defect, in Dr. C's eyes; but he ought to recollect that such a minute examination could hardly be expected in a remote part of the country. The following paragraph appears in the chemical part of the examination.

"In analyzing the suspected fluid by the different tests used for detecting the presence of arsenic, we have not been able to discover any marks or trace of that mineral; but, from our not having the chemical apparatus necessary for a complete trial of the more delicate tests, it is recommended, provided it can be legally done, that a still farther trial should be made by some practical chemist, in ascertaining the presence of poison; and, for this purpose, a part of the fluid has been reserved." 288.

This specimen was sent to Dr. C., and analyzed by him three months after the man's death. The result was quite satisfactory, and as the quantity detected was far more minute than was ever unequivocally detected before, in any judicial case, he needs no apology for publishing the process of analysis. We shall give it in the Doctor's own words:—

"The substance transmitted was a thick, slightly viscid, putrescent, dirty-gray fluid, with a few floating coagula; and it amounted to an ounce and a quarter. It was poured into a porcelain vessel, diluted with the washings of the phial, boiled briskly for fifteen minutes, and thus changed into a transparent liquid, with dirty ash-gray flocks suspended in it. The solid matter being separated by filtration, a clear fluid was obtained, of a very pale, straw-yellow colour.

"A small portion of this fluid was tested with lime-water, and the ammoniacal nitrate of silver. Lime-water caused a very scanty, dirty grayish-white, floculent precipitate. The ammoniacal nitrate of silver caused a copious, dirty grayish-white, pulverulent precipitate, without the slightest tint of yellow.

"As the arsenic, if present at all, evidently existed in very minute proportion, and the fluid was so composite as to render the foregoing tests inconclusive; instead of wasting any more of it upon trials with the other liquid reagents, the whole of the remainder, with the washings of the filter, was acidulated with acetic acid, and subjected for fifteen minutes to a stream of sulphuretted hydrogen gas. It acquired, in consequence, a deeper yellow colour, and muddy appearance; which, when the excess of sulphuretted hydrogen was driven off by ebullition, gave place to a considerable precipitate, of a pale lemon-yellow colour, and composed partly of fibrous flocculi, partly of very minute, brilliant scales.* The presumption that arsenic existed in the fluid was thus strong.

"Next morning the precipitate having fallen down, the supernatant fluid was withdrawn with the pipette, and its place supplied with distilled water. The brilliant scales then became still more distinct. The operation of subsidence and affusion being repeated the same day, the precipitate was thrown upon a filter; and next morning, when the water had passed through, the filter was compressed, and partially dried between folds of bibulous paper. The moist precipitate was then removed, dried in fragments with a gentle heat, dropped into the bottom of a tube of this shape and size,†

* "This appearance of brilliant scales is not often produced when the quantity is small. The operator will have the best chance of obtaining it, if the excess of gas be driven off by a very gradually increasing heat.

† "This form of tube I borrowed from a paper by Berzelius, in Dr.

and covered with a little black flux, which was dropped through a paper tube, so as not to soil the glass. The whole material just filled the ball at the end of the tube. Heat was then cautiously applied with a very small spirit-lamp flame. Some water disengaged at first, was removed with a roll of filtering paper. On the farther application of heat, which was raised to full red, a distinct crust was sublimed into the narrow part of the tube. It was smooth, bluish-gray, and brilliant, like polished steel externally; crystalline, sparkling, and iron-gray internally, like the fracture of fine steel.

"The proof of the existence of arsenic was thus decisive. For no other known substance can yield, with sulphuretted hydrogen, a yellow precipitate, from which such a metallic crust can be sublimed. But as I was fully sensible of the influence which the foregoing analysis would have on the issue of the trial, I resolved to leave nothing undone to establish the nature of the crust precisely. For this end I subjected it to a test of great delicacy, elegance, and facility of application, and vastly superior in every respect to the supplementary test recommended in my former paper. It was suggested to me by my friend Dr. Turner, and, so far as I know, has not yet been made public. The portion of the tube containing the flux being removed by melting and drawing it out, the crust was driven up and down by the spirit-lamp flame. At first it kept its polish and iron-grey colour. But at length it was all converted into little detached crystals, white, translucent, of adamantine lustre, and evidently shewing triangular facets under a microscope of four powers. These crystals, I need hardly add, were octaëdral crystals of oxide of arsenic. The tube was weighed before and after the crystals were washed out with distilled water; and their weight thus proved to be one-twentieth of a grain.

"The solution, amounting to a dram, was divided into three portions, and subjected to the ammoniacal sulphate of copper, the ammoniacal nitrate of silver, and sulphuretted hydrogen. These tests all gave pointed indications; but it required very cautious management to make them distinct.

"This analysis left no doubt that the deceased had taken arsenic; and as the symptoms and mode of death, as well as the morbid appearances, corresponded with what arsenic is known to produce, I could not hesitate to ascribe his death to its administration." 292.

For various chemical remarks on the foregoing analytical process, we must refer to the paper in the original work. We shall, therefore, proceed to the second case of poisoning by arsenic.

*Case* 2. E. O., a girl, fourteen years of age, was a servant in a fa-

---

Brewster's Journal for January last. The paper contains an account of a method for detecting minute quantities of arsenic. It is very nearly the same with that I have recommended, except that, when the quantity of sulphuret on the filter is too minute to be collected, the Professor recommends an additional and very complicated process, for converting the sulphuret of arsenic into the arseniate of lime, before employing the test of reduction. A much better plan, when the quantity is so very minute, is to throw the liquid and precipitate into a small glass funnel, corked at the bottom. The precipitate having subsided, the greater part of the fluid may be withdrawn with the pipette, and the remainder evaporated, by putting the funnel at the side of a fire. The sulphuret may be then picked out, without a particle being lost. Berzelius's plan is far too complex for any other than an expert analyst."

mily, which she had several times left, in disgust, but was always brought back again by her friends. The last occurrence of this kind was a week before her death. She was occasionally dull and melancholy, but not particularly so on the morning of her illness—on the contrary, she was more in spirits than usual. She had habitual good health. At two in the afternoon, after being seen a few minutes previously in good health, she was found by her mistress in bed, yawning and retching, and complaining of head-ache, sickness, and pain in the bowels. She also complained of thirst, and a sense of coldness. At half-past five, the pain in her bowels continuing, her mistress gave her some whiskey-punch—the only thing she took during her illness. At six, she became blue in the face, and seemed fainting. A surgeon was called, but before he arrived she had breathed her last. Her illness continued about five or six hours.

One or two evenings before this tragic event, a girl, answering her appearance, went to a chemist's shop, and asked for a penny-worth of arsenic to kill bugs. It was given to her (two drachms) without any more questions, and without even putting a label on the paper. There were no bugs in the house. The body was buried three days after death; but was disinterred the following day, on judicial inquiry, and examined by Dr. Christison himself, and Mr. Watson. The dissection and chemical examination we deem it proper to give in the words of the author:—

"There was not any outward appearance worthy of remark, except that the countenance had a very calm expression, and that the skin was nowhere livid. The latter fact I specify, because some absurdly enough imagine that lividity is invariable after poisoning with arsenic.

"In the head, the only thing of note was a greater turgescence than usual, of the veins of the superficial and ventricular membranes of the brain,—not so great, however, as to amount decidedly to morbid congestion.

"The organs in the chest were perfectly natural in appearance.

"The veins, as seen on the outer surface of the stomach, were somewhat gorged. The stomach, after being secured by a ligature at each end, was taken out, emptied of its contents, and slit up. The contents were reddish-coloured and limpid, with a few albuminous-like flakes, but no white powdery particles; and they had a smell of some vinous or spirituous liquor. They measured 11½ ounces. The inner membrane of the stomach was every-where mottled with small coalescing patches, of an exceedingly faint cherry-red colour, confined to the villous coat; and around the upper orifice there were a few more distinct and very minute scarlet specks. The villous membrane could be peeled off with facility.

"The intestines, viewed externally, appeared much injected with dark-blood. Internally, the villous membrane had the same appearance as in the stomach; but the shade was even paler. They contained hardly any contents; the small intestines particularly were quite empty.

The contents of the stomach, as well as the stomach itself, were taken to Edinburgh to be analysed; but we were not permitted to carry away the intestines, and it was not without difficulty that we obtained even the stomach. In fact, throughout the whole examination, we were much obstructed by the friends, and a surgeon who accompanied them; and the wrangling in which we were thus involved, coupled with a deficiency in the warrant, led to the neglect of many points which it was of consequence to ascertain.

"Besides the stomach and its contents, we obtained for analysis, from one of the friends present at the inspection, a white powder found in a box belonging to the deceased.

"The analysis of these three articles was performed by myself, and most of the essential steps were taken in Mr. Watson's presence.

"1. I commenced with the powder. It was heavy for its bulk, and weighed 26 grains. A small quantity, subjected with black flux to the process of reduction in a glass-tube, gave an abundant crust, possessing all the physical properties of metallic arsenic, (the garlic smell included), and yielding crystals, with triangular facettes, when heated in the way formerly mentioned. The powder, therefore, contained a large proportion of arsenic. Another quantity was heated on an iron-plate; it sublimed at a low temperature in white fumes, and left a barely perceptible residue. The powder was, therefore, very nearly pure oxide of arsenic.

"2. The contents of the stomach were evaporated to a third of their volume, and filtered. The fluid, which had a very pale straw-yellow colour, gave a scanty, grayish-white cloud, with lime-water; a copious dirty, grayish-white precipitate, with the ammoniacal nitrate of silver; and underwent no change with sulphuretted hydrogen. These tests, therefore, did not give any indication of the presence of arsenic. The remainder was evaporated to the volume of half an ounce, when it acquired a brownish-red colour. In this state, when treated as in the former case with sulphuretted hydrogen, it gave an evident brownish-yellow, flocculent precipitate; which, however, was so scanty, that, before proceeding farther, I thought it right to wait for the result of the analysis of the stomach itself.

"3. Accordingly, the stomach was cut into small fragments, and boiled briskly, for half an hour, with a pint of water, in a porcelain basin, glazed with porcelain. The larger masses of solid matter were then removed, and the fluid filtered. One-half of it passed through in two days, and was subjected to analysis by sulphuretted hydrogen, after previous concentration to the volume of half an ounce. A dirty lemon-yellow precipitate was thus obtained, amounting apparently to twice the volume of that procured from the contents. As I was sure I had now got enough to try the test of reduction, I mixed the two precipitates, and, following the course pursued in the former case, I obtained precisely the same results. The quantity of oxide eventually formed I did not weigh; but it was certainly not greater than a twentieth of a grain. The whole quantity of oxide, including what was in the contents, and in that part of the decoction of the stomach which was not filtered, could not have surpassed a fifteenth of a grain.

"The analysis, together with the symptoms, left no doubt that the girl had died of poisoning with arsenic.

"This case presents several particulars, both for comment and instruction. I shall be very short, however, in my remarks, considering the detailed account given of the former.

"In the *first* place, the total absence of morbid appearances in the stomach, is a rare fact in the history of poisoning with arsenic. It is not, perhaps, very generally known; yet it has been observed before.* It proves,

---

* "I may refer to the following cases, as the most pointed of the kind, which I have met with in the course of my reading.

"Etmuller, Ephemerides Acad. Cæsareo-Leopoldinæ 1715. Obs. 126.

"Laborde, Journal de Médécine, t. lxx.

that the arsenic had remained but a short time in the stomach during life; and therefore accords with the account given of the girl's illness, which probably did not last above five hours. In shortness of duration this case corresponds with every other hitherto published, in which the stomach was found in a natural state; and it likewise corresponds with them in the symptoms having been by no means well marked.

"*Secondly*, The want of morbid appearances, together with the almost total absence of arsenic in the stomach, and the fact that the girl was fasting at the time she took it, renders it not improbable that it had been taken in solution. It is not easy to account otherwise for so small a quantity being found in the stomach, when she vomited so little, and had so short an illness. It may be objected, that this method of taking arsenic is very rarely resorted to, and would not likely occur to an ignorant girl. But, on the other hand, she had several times taken laxative salts against her mistress's wish, for the purpose of making herself ill; and she might consequently be led to use the arsenic in the same way as the salts.

"*Thirdly*, with regard to the proof of poisoning, it is clear that the symptoms and appearances after death, while they do not contradict it, hardly uphold it even by presumptive evidence; that, in short, the case, in a legal point of view, hung entirely on the chemical evidence.

"*Fourthly*, By any other method of analysis, particularly by those practised in Britain, there is good reason for believing that the poison would not have been detected. I need scarcely point out, therefore, how important it is that the proposed method should be minutely examined, and become generally known. In my own hands it has hitherto proved susceptible of universal application, and of yielding the most positive proof in the most unlikely circumstances; and I am satisfied, that, in the hands of another, it will prove easy of application, though he should be neither a very adroit, nor a very practised operator.

"It is not too extravagant to assume, that, had the process been earlier known, several late judicial cases would have been made perfectly clear, which, for want of good chemical evidence, have excited a considerable ferment in the public mind, and thrown our courts into doubt and embarrassment; and that henceforward we shall not often hear of criminals escaping from justice, because the medical witness could not discover the poison.

"I have said that I have hitherto found this method susceptible of universal application. I can foresee the possibility of one difficulty, however. If the fluid containing the arsenic is ropy and viscous, even after being boiled and acidulated with acetic acid, the sulphuret thrown down may be mingled with so much animal matter, that the metallic sublimate is not distinctly formed, on account of the great quantity of empyreumatic matter suddenly projected along with it. This has happened to me once only out of at least a hundred experiments. In that case an addition must be made to the process. The criterion by which we may know that the additional process is required, is the colour of the precipitate. If the colour is lemon-yellow, or pale brownish-yellow, it is unnecessary; if it is cream-white, the simple process will probably fail. The plan I should prefer is a modification of that proposed by Berzelius, and of the method followed by Roloff (p. 279), for determining the quantity of arsenic in the sulphureous preci-

---

"Jaeger, de Effectibus Arsenici. Diss. Inaug. Tubingæ 1808, p. 23-39.
"Chaussier, Orfila Toxicologie Générale, i. 155. 1ere Edition.
"Medical and Physical Journal, xxxiv.

pitate. Treat the precipitate with nitric acid, and thus convert the sulphuret into sulphuric and arsenic acids; dissolve and filter; neutralise with ammonia, and throw down the arsenic and sulphuric acids with nitrate of lead; reduce the precipitate, which contains arseniate of lead, in the usual way.

"*Lastly*, The unhappy fate of this poor young girl, who was only fourteen, and looked two years younger, taken along with the frequency of similar accidents, will naturally raise our astonishment, that our country should be so long in following the example of the Continental governments, by enacting police regulations for the sale of poisons, particularly in great towns. It may be easily imagined what abuses prevail, when arsenic could be got by such a purchaser. I am far from reflecting on the apothecaries of this town generally: I know that many of them never sell the common poisons but when they know the buyer. They will of course remember, that penal statutes are never intended for the honest and conscientious part of society." 307.

The second part of Dr. Christison's paper, contains commentaries on the symptomatological evidence of poisoning by arsenic.

Till medical jurisprudence had arrived at a certain degree of cultivation, much importance was naturally attached to certain symptoms which were supposed to be distinctive of the operation of poisons, especially of arsenic. Indeed, instances are not very rare, where medical witnesses have not scrupled to give pretty confident opinions based solely on the symptoms. The confidence in this evidence has been gradually decreasing, and medical jurists seem to have now passed to the other extreme, and maintain that symptoms can never lead to more than suspicion, or an opinion in favour of probability. As a general rule, this scepticism is considered proper by Dr. C. but not always to be adhered to. Three instances have come to his knowledge, within the last five years, "in which the general rule either was, or might have been, departed from." This departure from the rule appears allowable chiefly in the very cases where it is most essential—namely, where there is least chance of the poison being detected by chemical analysis. The particulars of the following case were referred to Drs. Duncan and Christison, for their opinions.

Charles Munn, a butcher, was indicted in 1824, for the double crime of murdering by poison, and procuring abortion. The purely moral evidence is partially omitted, as not essentially connected with the medical enquiry. It was circumstantial, but strong, and a part of it was so mixed up with the medical evidence, as to be requisite for its elucidation. The deceased became pregnant by the prisoner in the spring of 1823, and quickened in August. On two occasions, he tried to buy, and indeed did procure poisons, for the alleged purpose of killing rats. The poisons were tartar emetic, or arsenic, or both. On the evening of the 1st September, he met the girl alone, and persuaded her to take two tea-spoonfuls of a white powder in some water, saying it would shew whether or not she was pregnant. Soon after taking it, she got sick, and began to vomit. This last symptom recurred frequently during the night, and next morning she was unable to go to work till after breakfast. On the 4th September, the prisoner again met her, and prevailed on her to take another drug, in the form of a cake, which she swallowed with much difficulty. In half an hour she was attacked with sickness as before; and, from this time till the 9th, when a surgeon was called, the prominent symptoms were vomiting, purging, (sometimes of blood) and soreness of the mouth, throat, stomach, and bowels, with hoarseness and oppressed breathing. When the surgeon saw her on the 9th, the pulse was 120, full, soft, and regular—skin hot and dry—tongue parched and excoriated—throat spotted with little white ulcers—voice hoarse and feeble—breathing hurried and laborious—deglutition difficult—belly round, swelled, and painful, but not tender to the touch—great sense of exhaustion. On the 10th another surgeon was called in, and his account of the symptoms was very accurate and circumstantial.

"The pulse was 120, and throbbing; the breathing difficult; the tongue red and parched; the gums tender and shining, without salivation; she had soreness in the throat, descending along the gullet into the chest, and there was an excoriation on each side of the uvula, a white ulcer on the left tonsil, and redness and tenderness of the rest of the throat and back of the palate; she swallowed with such difficulty that a small quantity of any liquid caused violent gasping, hurried cough, and much pain; she complained farther of sickness and dull pain, and tenderness in the stomach and bowels; and likewise of pains in the feet and legs. The vomiting continued to recur throughout the day. In the evening the fever had increased; the pains of the feet and legs were also more severe; and she complained of soreness and tenderness of the labia pudendi; though in neither situation any unnatural appearance could be discovered.

"On the 11th and 12th there was not any material alteration in the symptoms, except that, on the evening of the former, she had some delirium; that on both days the fever lessened considerably in the morning and increased at night; and that the pains had abated a little. About midnight of the 12th she was taken ill with labour-pains, and in three hours brought forth a still-born fœtus, weighing nineteen ounces, and apparently between the fifth and sixth month. The delivery was difficult and distressing; and although there was no flooding, she was so feeble, that the midwife did not expect her to survive. The motions of the child had ceased on the 6th." 312.

On the 13th she was easier, and the vomiting ceased, and for the next three days the fever continued, with morning remissions and evening exacerbations, all the pains having abated, except those in the feet and legs. On the 16th she was so much better that she was able to undergo a judicial examination. For fourteen days she improved rapidly, but still complained of want of power in her feet and hands, with such severe pain in them as required opium to make her sleep. On the 1st October the prospect changed. The fever returned, and assumed a low type; and she died on the 19th of the same month—forty-five days after taking the second drug administered by the prisoner. The body was examined the day after death.

"The membranes of the brain were red and vascular, and serum was effused between the arachnoid and pia mater. The points of blood seen on cutting the brain were more numerous than usual. The ventricles contained a little serum.

"The sac of the pericardium also contained a little serum. The heart was sound. The lungs adhered to the chest by firm adhesions, but were themselves healthy.

"The stomach was natural in appearance outwardly. It contained a little curdled milk. Its villous coat had a slight red colour at various points; and at the pyloric end of the lesser curvature it was very vascular, and of a dark-red hue. The redness was a diffused, brownish-red blush, not referrible to vascularity by the naked eye, and disposed in spots as big as a crown-piece. The small intestines were every where red and vascular; and at one part the gut was thickened along three inches of its course. The colon was red and vascular, where it lies over the lower end of the stomach. No ulceration was visible in the whole alimentary canal. The uterus was large, but healthy, except at the mouth, where it was very hard, and of a dark-red colour. The vagina was natural in appearance; and so were the liver, spleen, kidneys and bladder." 314.

We cannot follow our author through all his comments on this case, which is very complex in a medico-legal point of view, though Dr. C. thinks it is by no means doubtful, when the questions involved in it are clearly separated and duly considered. Dr. C. conceives that there cannot be a doubt that the miscarriage was owing to the violent irritation of the system, or consequent debility, or both together—these last being the result of poison and not natural disease. At the same time, there is no chemical proof of poison having been administered—nor can the appearances found on dissection be taken into account—the cause of death being much more obscure than the cause of the early symptoms. All circumstances duly considered, Dr. Duncan and Dr. Christison came to the conclusion that it was *very probable* that the primary disorder under which the girl laboured, between the 4th and 30th September, was caused by some acrid poison.

The question, "what was the kind of poison administered?" leads our author to make many interesting observations on the symptoms which attend the ingurgitation of particular poisons. These observations, indeed, are of such importance that we shall give them in the Doctor's own words.

"There are some poisons, the symptoms of whose action are generally so characteristic, that an experienced person cannot confound them with any natural disease, or with any other poison. Others possess this characteristic action more rarely. Of the first kind are *oxalic acid* and *strychnia;* of the second, *corrosive sublimate* and *arsenic.*

"Few opportunities have hitherto occurred, for ascertaining correctly the symptoms of poisoning with *oxalic acid* in those most frequent cases in which it proves fatal within an hour. The instances, however, which have been accurately observed, coupled with what is known of its effects on animals, lead to the conclusion that it generally causes a very sour taste,—a sense of burning along the throat and gullet in the act of swallowing,—acute burning pain in the stomach immediately afterwards,—then violent vomiting,—next sudden failure of the pulse and strength,—and death in ten, twenty, thirty, or sixty minutes, sometimes under a state of pure and rapidly increasing faintness, sometimes at the close of one or more attacks of violent tetanic spasm.* Such a succession of symptoms, within such an interval, cannot be caused by any other poison†, or by any natural disease or combination of diseases, with which I am acquainted.

" *Strychnia* is another poison which causes almost invariably symptoms quite characteristic of its action. If, within a few minutes after taking an intensely bitter substance, a person is attacked with pure and violent tetanus, recurring in frequent fits, and proving fatal in five, ten, twenty, or sixty minutes, I cannot conceive it possible to draw any other inference, than that death has arisen from poisoning with strychnia, or some of the poisons which contain it. These symptoms are almost invariable, at least in the case of strychnia itself. There is a way, indeed, in which this alkaline principle might be administered, so as to act differently, and to render it difficult to form the foregoing opinion. For obvious reasons I shall not mention it. It could only be tried with success by one very well acquainted with the properties of the poison. On the whole, therefore, a decided opinion may be drawn in almost every instance from symptoms only ; and this is a point of consequence, because other medical evidence will seldom be attainable, and nothing but the difficulty of procuring the drug keeps it now out of the hands of the poisoner.

" *Corrosive sublimate* is one of the poisons that produce characteristic symptoms only on some occasions. Several cases are on record, in which it has caused a strong metallic and astringent taste,—a sense of corrosion and burning in the throat or gullet, or both, in the act of swallowing,—acute burning pain in the stomach and belly soon afterwards,—speedy, violent, and often bloody vomiting,—afterwards purging of the same description ; and on the second or third day, or a little later, more or less salivation, with fetor of the breath,—ulcers of the gums,—dropping out of the teeth, and even gangrene of the mouth,—all the signs, in short, of true mercurial salivation. In the event of such a case occurring in a criminal court, I cannot see how a witness could avoid giving the opinion, that corrosive sublimate, or some soluble salt of mercury, had been taken." 320.

Dr. C. endeavours to illustrate the foregoing observations by reference to a trial which took place not long ago, on a medical gentleman in Sunderland. But as the accused was acquitted, we do not think it right to enter at all on such a delicate subject.

Dr. C. next endeavours to shew that arsenic is another poison which may, at times, produce symptoms such as cannot originate from any other cause.

" Arsenic, when it does not prove fatal, or only after a-week or upwards,

---

* " This last symptom has not been noted in any of the cases hitherto published, of poisoning in the human subject. As in similar circumstances Dr. Coindet and I found it to be almost invariable in our experiments on animals, I should have felt surprise at the difference, did I not consider, that very few of the cases have been seen at the point of death by a medical man, and that the symptom is almost a momentary one. (See our paper in the Edinr. Med. and Surg. Journal, April 1823.)"

† " The mineral acids, which, in many respects, resemble oxalic acid in their action, do not cause tetanic spasms, and never prove so speedily fatal. Of the fifty-six cases reported by Tartra, in his excellent work on poisoning with nitric acid, none proved fatal in a shorter period than six hours. (Traité de l'empoisonnement par l'Acide Nitrique, p. 160.)"

often causes a singular complexity of symptoms, denoting inflammation, or at least violent irritation in the throat, gullet, stomach, and intestines; in the windpipe; in the mucous membrane of the eyes and nose; in the urinary bladder, urethra and vagina; in short, in the whole mucous surfaces of the body. In such cases, too, there are occasionally eruptions of the skin; sometimes petechial; sometimes measly; sometimes miliary. And more frequently, the inflammatory symptoms are accompanied or succeeded, on the one hand, by complete or incomplete palsy of one or more of the extremities, and sometimes, too, racking pains in the palsied parts; or, on the other hand, by frequent fits of epilepsy, or by both together. No medical jurist could doubt that arsenic had been given, if he met with such a conjunction of disorders. They cannot be produced by any other poison, so far as our knowledge goes; and as little can they be caused by any natural disease, or any union of diseases ever known. It is true, that such a union of symptoms from natural causes is conceivable. But if it is so, it could never take place without its origin being clearly pointed out by collateral circumstances." 324.

The rule now laid down, Dr. C. thinks, might have been applied to two trials which happened in Britain not long ago—one of which was the poisoning of a family by Eliza Fenning. Perhaps a thorough investigation of the subject would lead to conclusions not materially different, where arsenic proves suddenly fatal. In these, death generally takes place within two days and a half, the symptoms indicating inflammation of the stomach, intestines, and even the œsophagus. The affection of the throat precedes the vomiting—the latter is often bloody, from time to time—and, after death, there are found, either in the sto-stomach or intestines, marks of inflammation, or great turgescence of vessels.

There are three diseases, whose effects are somewhat similar—gastritis, cholera, and what are called, spontaneous perforations of the stomach. Acute gastritis is rare in this country—especially such a form as would destroy life in the abovementioned period. In respect to cholera, whose symptoms are so similar to those occasioned by arsenic, Dr. C. thinks that a diagnosis may be drawn between the two.

"1. In cholera, the vomiting is never bloody. Even in the epidemic cholera of the East, it is not; at least no such symptom is mentioned in the reports lately published by the three Indian Presidencies. 2. The sense of burning in the throat and gullet, at all times a rare symptom in cholera, always *succeeds* the vomiting. 3. I doubt whether the cholera of this part of the country ever proves fatal in so short a time as two days and a-half. At least according to the experience of all my acquaintances it does not." 327.

In this Dr. C. is wrong. Two distinct cases of cholera proving fatal within 36 hours, came under our own notice during the last summer in London; and we heard of two or three more. When cholera proves fatal at all, in this country, it is within the period of two or three days. We may appeal to Sydenham who, in his description of this disease, says it sometimes "frightens the bye-standers and kills the patient in 24 hours."

As to the spontaneous perforations of the stomach, "if the continental pathologists are correct, the symptoms closely resemble those of acute poisoning with acrids." The vomiting, however, is never bloody—and the appearances in the stomach are very peculiar. But it is to be remembered that blood is not always thrown up in poisoning by arsenic.

The last question discussed by our author relates to the cause of the girl's death, in the case related above. The surgeons of Rothsay have, in Dr. C.'s opinion, taken an erroneous view of the subject, even after they had seen the report of Drs. Duncan and Christison. They were of opinion that, "if the poison was the cause of the girl's first illness, it was also the cause of her death." In this we certainly cannot agree with them.

That there was an inflammatory affection of the mucous membrane of the stomach and bowels, between the 4th and 16th September, there can be little doubt; but that the last illness was a fever, not necessarily connected with any such local inflammation, is highly probable. Hence her death could not, with safety, be attributed either to the direct or indirect operation of the poison.

"On the contrary, the irritant poisons are remarkable for the steady progress of their symptoms. There may be a few remissions and exacerbations; but otherwise the case goes on very uniformly till the person dies or recovers. They are likewise remarkable for the homogeneousness of their symptoms: That is, their character as to kind remains much the same from first to last. Some of them, indeed, do not come under this observation. For many which have been classed with the irritants are really narcotico-acrids; and in their case the symptoms of irritation often give place to those of nervous derangement, such as coma, palsy, epilepsy, and the like. But I am not aware of the existence of any poison, which, after it has caused inflammation of the alimentary canal, and that has subsided, can then excite a protracted general fever, unaccompanied with local disease.

"This remark applies with peculiar force to the poison believed to have been given in the present case. Among the numerous examples of poisoning with arsenic, which are to be found in authors, neither Dr. Duncan nor I could remember any one at all parallel. As it was only from the occurrence of parallel cases, that an opinion in favour of death by poison could be drawn, we were necessarily led to agree, that the real cause of death could not be specified with any certainty." 330.

The prisoner Munn, was found guilty of the crime of procuring abortion by administering poison; but the charge of murder was found not proven.

Here we have brought the analysis of this long paper, (occupying 55 pages of the Transactions) to a close. Dr. Christison appears to be cultivating with zeal and talent, a very important branch of medical jurisprudence, and we cannot but hope that he will persevere till he has thrown light on some of the dark paths of toxicology.

## Figures and Tables

**Figure f1:**